# Surfen and oxalyl surfen decrease tau hyperphosphorylation and mitigate neuron deficits in vivo in a zebrafish model of tauopathy

**DOI:** 10.1186/s40035-018-0111-2

**Published:** 2018-03-16

**Authors:** Seyedeh Maryam Alavi Naini, Constantin Yanicostas, Rahma Hassan-Abdi, Sébastien Blondeel, Mohamed Bennis, Ryan J. Weiss, Yitzhak Tor, Jeffrey D. Esko, Nadia Soussi-Yanicostas

**Affiliations:** 1 PROTECT, Inserm, Université Paris Diderot, Sorbonne Paris Cité, Paris, France; 2 Institut de Biologie Paris Seine-Laboratoire Neuroscience Paris Seine, Inserm UMRS 1130, CNRS UMR 8246, UPMC UM 118, Université Pierre et Marie Curie, Paris, France; 3 Cadi Ayyad University, Marrakesh, Morocco; 4 Department of Chemistry and Biochemistry, University of California, San Diego, La Jolla, CA USA; 5 Department of Cellular and Molecular Medicine, University of California, San Diego, La Jolla, CA USA

**Keywords:** Tauopathy, Zebrafish, Alzheimer’s disease, Tau protein, Tau hyperphosphorylation, Surfen, Oxalyl surfen, Heparan sulfate

## Abstract

**Background:**

Tauopathies comprise a family of neurodegenerative disorders including Alzheimer’s disease for which there is an urgent and unmet need for disease-modifying treatments. Tauopathies are characterized by pathological tau hyperphosphorylation, which has been shown to correlate tightly with disease progression and memory loss in patients suffering from Alzheimer’s disease. We recently demonstrated an essential requirement for 3-*O*-sulfated heparan sulfate in pathological tau hyperphosphorylation in zebrafish, a prominent model organism for human drug discovery. Here, we investigated whether in vivo treatment with surfen or its derivatives oxalyl surfen and hemisurfen, small molecules with heparan sulfate antagonist properties, could mitigate tau hyperphosphorylation and neuronal deficits in a zebrafish model of tauopathies.

**Results:**

In vivo treatment of Tg[HuC::hTau^P301L^; DsRed] embryos for 2 days with surfen or oxalyl surfen significantly reduced the accumulation of the pThr181 tau phospho-epitope measured by ELISA by 30% and 51%, respectively. Western blot analysis also showed a significant decrease of pThr181 and pSer396/pSer404 in embryos treated with surfen or oxalyl surfen. Immunohistochemical analysis further confirmed that treatment with surfen or oxalyl surfen significantly decreased the AT8 tau epitope in spinal motoneurons. In addition, in vivo treatment of Tg[HuC::hTau^P301L^; DsRed] embryos with surfen or oxalyl surfen significantly rescued spinal motoneuron axon-branching defects and, as a likely consequence, the impaired stereotypical touch-evoked escape response. Importantly, treatment with hemisurfen, a surfen derivative devoid of heparan sulfate antagonist activity, does not affect tau hyperphosphorylation, nor neuronal or behavioural deficits in Tg[HuC::hTau^P301L^; DsRed] embryos.

**Conclusion:**

Our findings demonstrate for the first time that surfen, a well-tolerated molecule in clinical settings, and its derivative, oxalyl surfen, could mitigate or delay neuronal defects in tauopathies, including Alzheimer’s disease.

**Electronic supplementary material:**

The online version of this article (10.1186/s40035-018-0111-2) contains supplementary material, which is available to authorized users.

## Introduction

Tauopathies comprise more than 20 neurodegenerative diseases including Alzheimer’s disease (AD), frontotemporal dementia (FTD), Pick’s disease, progressive supranuclear palsy (PSP) and other related disorders. Tauopaties are characterized by accumulation of hyperphosphorylated isoforms of the microtubule-associated tau protein in brain forming distinct inclusions [1].

We have recently shown that in vivo depletion of Hs3st2, an enzyme involved in 3-*O*-sulfation of heparan sulfate chains and predominantly expressed in neuronal cells, significantly decreased tau hyperphosphorylation and partially rescued neuronal and behaviour defects in transgenic Tg[HuC::hTau^P301L^; DsRed] zebrafish embryos [2]. Tg[HuC::hTau^P301L^; DsRed] embryos display key features of tauopathies, such as tau hyperphosphorylation [3]. These findings suggest that inhibition of heparan sulfate-related activities could have beneficial therapeutic effects for tauopathies. Here, we have tested the hypothesis that treatment of Tg[HuC::hTau^P301L^; DsRed] zebrafish embryos with small molecules displaying heparan sulfate antagonist properties could mitigate pathological tau hyperphosphorylation and rescue the induced neuronal and behavioural deficits.

Glycosaminoglycan (GAG)-protein interactions have long been recognized as therapeutic targets in various disease conditions such as cancer, inflammation and AD [4, 5]. Inhibition of harmful processes mediated by endogenous GAGs, by treatment with exogenous GAGs or GAG mimetics, has been developed as a therapeutic strategy in Alzheimer’s disease [6–8]. Surfen (1,3-bis (4-amino-2-methylquinolin-6-yl) urea) is a quinolone-based low MW derivative, which was initially developed for the production of depot insulin for diabetic patients [9]. This well-tolerated substance was later shown to possess antibacterial, trypanocidal, and anti-inflammatory properties [10–12]. Of particular interest, surfen was later shown to exhibit heparin-neutralizing activity and the ability to antagonize heparan sulfate (HS)-protein interactions [5, 13]. As a first attempt to investigate the effects of surfen and its analogs, on tau hyperphosphorylation, we have examined whether surfen and two recently synthesized surfen derivatives, oxalyl surfen (*N*^1^,*N*^2^-bis(4-amino-2-methylquinolin-6-yl)oxalamide) and hemisurfen (1-(4-amino-2-methylquinolin-6-yl)urea) [14], could reduce tau hyperphosphorylation and alleviate neuron defects in vivo in Tg[HuC::hTau^P301L^; DsRed] zebrafish embryos.

## Results

### Surfen, oxalyl surfen and hemisurfen are well tolerated in zebrafish embryos

**Fig. 1:**
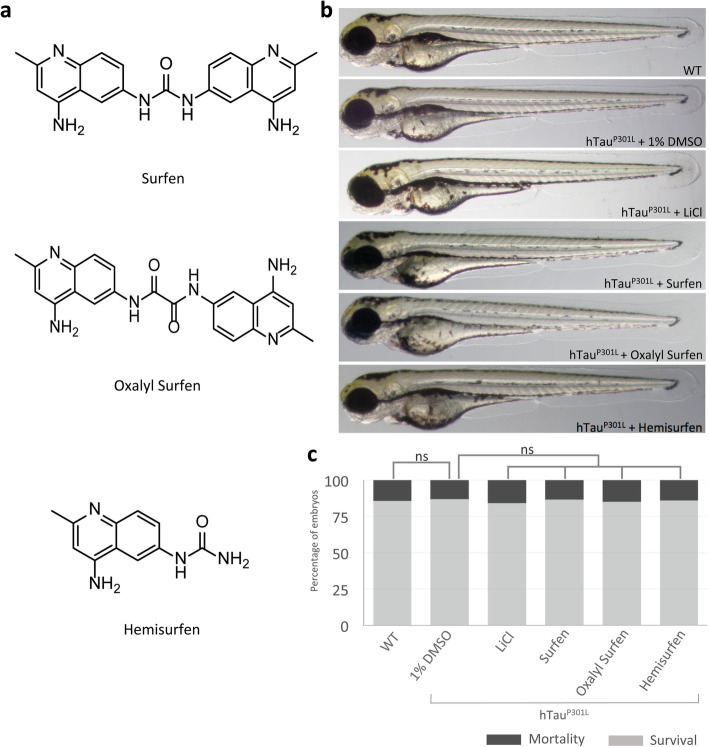
Surfen and oxalyl surfen are well tolerated by Tg[HuC::hTau^P301L^; DsRed] embryos. **a** Chemical structure of Surfen (1,3-bis(4-amino-2-methylquinolin-6-yl) urea), oxalyl surfen (N1,N2- bis(4-amino-2-methylquinolin-6-yl)oxalamide) and hemisurfen (1-(4-amino-2-methylquinolin-6- yl)urea). **b** Phenotypic analysis of 72 hpf wild-type (WT) and Tg[HuC::hTau^P301L^; DsRed] (hTau^P301L^) embryos incubated for 2 days in E3 medium containing 1% DMSO (hTau^P301L^ + 1% DMSO), 80 mM LiCl (hTau^P301L^ + LiCl), 3 μM surfen (hTau^P301L^ + surfen), 2 μM oxalyl surfen (hTau^P301L^ + oxalyl surfen) or 3 μM hemisurfen (hTau^P301L^ + hemisurfen), showed that embryonic development is not impaired by the treatments. Magnification × 40. **c** Survival rate of 72 hpf wild-type (WT) and Tg[HuC::hTau^P301L^; DsRed] (hTau^P301L^) embryos incubated for 2 days in E3 medium containing 1% DMSO (hTau^P301L^ + 1% DMSO), 80 mM LiCl (hTau^P301L^ + LiCl), 3 μM surfen (hTau^P301L^ + surfen), 2 μM oxalyl surfen (hTau^P301L^ + oxalyl surfen), or 3 μM hemisurfen (hTau^P301L^ + hemisurfen), demonstrated that embryonic mortality was not significantly increased by treatments (*n* = 250, *P* > 0.05, Student’s *t* test)

Initially, experiments were set up to analyze potential toxicity of the surfen analogs used in this study, (Fig. 1a). One set of 24 h post-fertilization (hpf) zebrafish Tg[HuC::hTau^P301L^, DsRed] embryos were incubated for 2 days in E3 medium containing 1% DMSO and surfen, oxalyl surfen or hemisurfen. As negative controls, age-matched wild-type and Tg[HuC::hTau^P301L^, DsRed] embryos were incubated for 2 days in E3 medium containing 1% DMSO. As positive control, age-matched Tg[HuC::hTau^P301L^, DsRed] embryos were incubated in E3 medium containing 1% DMSO and 1 lithium chloride (LiCl), a long known inhibitor of tau hyperphosphorylation [15]. For each agent, operational concentrations were defined as the highest concentration not inducing any visible morphological abnormalities, including heart rhythm and blood flow defects (Fig. 1b), nor any significant increase in embryo lethality (Additional file 1: Figure S1c). Operational concentrations (3 μM for surfen, 2 μM for oxalyl surfen, and 3 μM for hemisurfen) were then used for all subsequent experiments.

### Surfen and oxalyl surfen reduce tau hyperphosphorylation in vivo

**Fig. 2:**
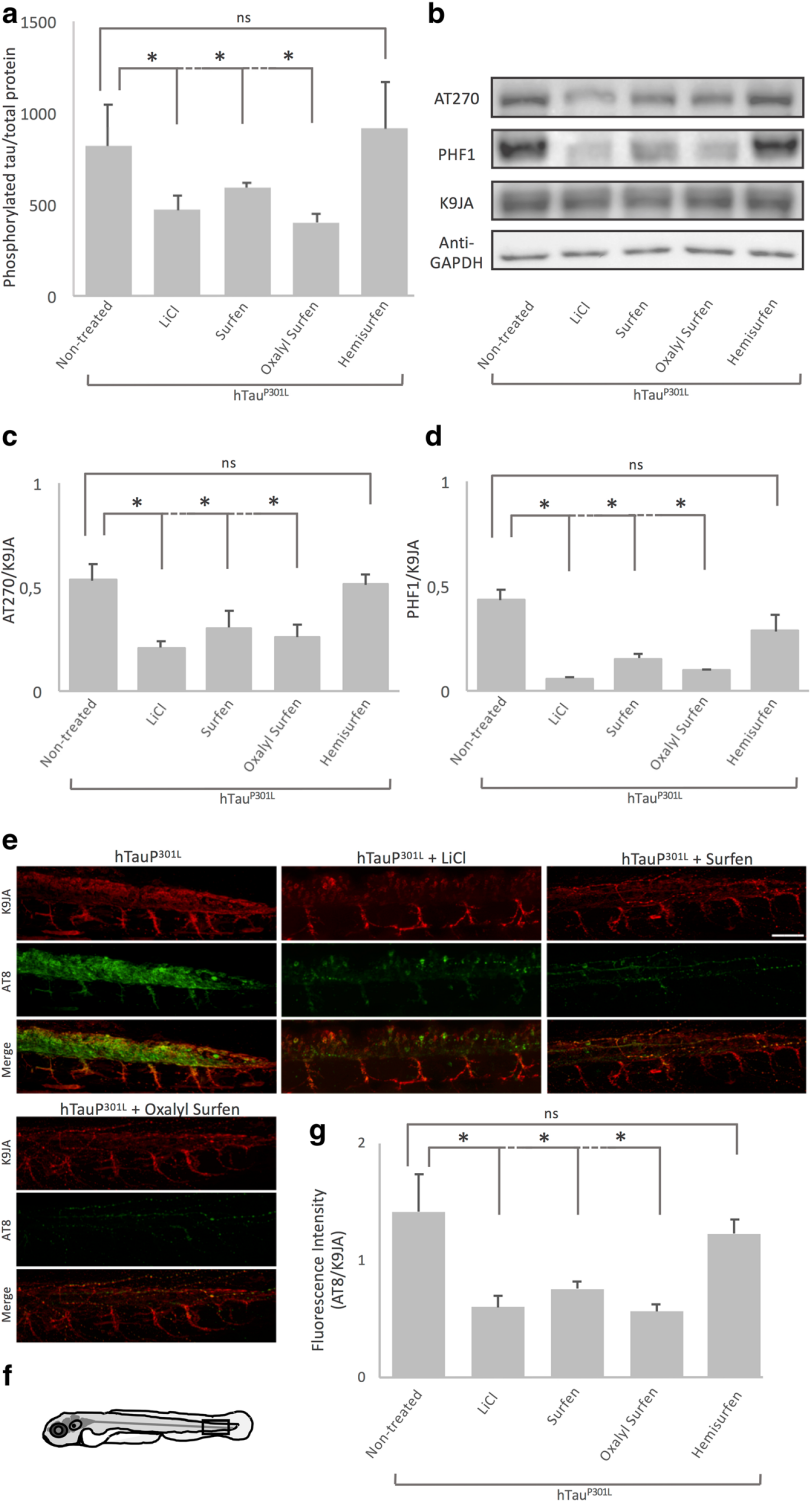
Treatments with surfen and oxalyl surfen, but not hemisurfen, decrease tau hyperphosphorylation in Tg[HuC::hTau^P301L^; DsRed] embryos in vivo. **a** ELISA quantification of pThr181 in 72 hpf Tg[HuC::hTau^P301L^; DsRed] (hTau^P301L^) embryos treated for 2 days in E3 medium containing 1% DMSO (hTau^P301L^ + 1% DMSO), 80 mM LiCl (hTau^P301L^ + LiCl), 3 μM surfen, (hTau^P301L^ + surfen), 2 μM oxalyl surfen (hTau^P301L^ + oxalyl surfen), or 3 μM hemisurfen (hTau^P301L^ + hemisurfen). The values were normalized to total human Tau protein recovered in each sample. pThr181 was significantly decreased in Tg[HuC::hTau^P301L^; DsRed] embryos treated with LiCl, surfen and oxalyl surfen, but not hemisurfen (* *P* < 0.05, ns: not significant, Student’s *t* test). **b** Western blot analysis of pThr181 (AT270 antibody), pSer396/pSer404 (PHF1 antibody) and total-Tau (K9JA antibody) in 1% DMSO 72 hpf Tg[HuC::hTau^P301L^; DsRed] (hTau^P301L^) embryos and in age-matched siblings treated for 2 days with 80 mM LiCl, 3 μM surfen, 2 μM oxalyl surfen or 3 μM hemisurfen, confirmed that treatments with surfen or oxalyl surfen, but not hemisurfen, markedly reduce tau hyperphosphorylation in Tg[HuC::hTau^P301L^; DsRed] embryos. **c** Densitometric analysis of AT270 on Western blots (represented in b) showing significant decrease of AT270/K9JA densitometric intensity ratio with LiCl, surfen and oxalyl surfen treatment, but not hemisurfen (*n* = 3, * *P* < 0.05, ns: non-significant, Student’s *t* test). **d** Densitometric analysis of PHF1 staining intensity on Western blots (represented in b) showing significant decrease of PHF1/K9JA densitometric intensity ratio with LiCl, surfen and oxalyl surfen treatment, but not hemisurfen (*n* = 3, * *P* < 0.05, ns: non-significant, Student’s *t* test). **e** Immunohistochemical visualization of total hTau protein (K9JA) and hyperphosphorylated tau epitope pSer202/pThr205 (AT8), and merged images of the two labelings (merge) in framed caudal area (**f**) in spinal cord of 72 hpf Tg[HuC::hTau^P301L^; DsRed] embryos and age-matched siblings incubated for 2 days with 80 mM LiCl (hTau^P301L^ + LiCl), 3 μM surfen (hTau^P301L^ + surfen), 2 μM oxalyl surfen (hTau^P301L^ + oxalyl surfen) or 3 μM hemisurfen (hTau^P301L^ + hemisurfen), confirmed the decrease of tau hyperphosphorylation following treatments with surfen and oxalyl surfen. **g** Quantification of AT8 fluorescence intensity. The AT8/K9JA fluorescence intensity ratio was significantly decreased following treatments with LiCl, surfen and oxalyl surfen, but not hemisurfen (*n* = 10, * *P* < 0.05, ns: non-significant, Student’s *t* test). Scale bar: 50 μm

As a first attempt to determine whether treatment with surfen, oxalyl surfen or hemisurfen could decrease tau hyperphosphorylation in vivo, we quantified tau pThr181 phosphorylation by ELISA assay. Treatment of 24 hpf Tg[HuC::hTau^P301L^; DsRed] embryos for 2 days with surfen or oxalyl surfen, decreased the accumulation of hyperphosphorylated tau by 30% and 51%, respectively (surfen and oxalyl surfen: *P* < 0.05), when compared to embryos treated with 1% DMSO (Fig. 2a). In contrast, no significant differences in pThr181 phospho-tau accumulation could be detected in embryos incubated with hemisurfen (*P* = 0.83, Fig. 2a). As expected, treatment with LiCl resulted in a 43% decrease in hyperphosphorylated tau when compared to embryos treated with 1% DMSO (*N* = 5, *n* = 250 (number of embryos); *P* < 0.05, Fig. 2a).

To confirm the ELISA results, we performed Western blot analysis with antibodies directed against either the pThr181 (AT270) or pSer396/pSer404 (PHF1) phospho-tau epitopes (Fig. 2b). Data showed a significant decrease in accumulation of both epitopes following treatment with surfen, oxalyl surfen and LiCl (*P* < 0.05). In contrast, hemisurfen did not significantly affect tau phosphorylation on AT270 nor PHF1 epitopes, (*P* = 0.42) and (*P* = 0.12) respectively (Fig. 2b, c, d).

To further investigate the effects of surfen and its derivatives on tau hyperphosphorylation, Tg[HuC::hTau^P301L^; DsRed] embryos were analysed by immunohistochemistry using the AT8 antibody directed against the pSer202/pThr205 phospho-tau epitope and the anti-total-tau K9JA antibody (Fig. 2e). In good agreement with both the ELISA results and Western blot analysis, a significant decrease in AT8/Total tau labelling intensity ratio was observed in the spinal cord of Tg[HuC::hTau^P301L^; DsRed] embryos treated with surfen, oxalyl surfen and LiCl (*P* < 0.05), but not hemisurfen (*P* = 0.3) (Fig. 2f). Interestingly, surfen or oxalyl surfen, decreases the somato-dendritic localization of hTau^P301L^ (Fig. 2e), a feature of tau pathology in AD and FTD [16]. As tau missorting is linked to tau hyperphosphorylation, the decrease in somato-dendritic tau might be a consequence of the decrease in tau phosphorylation. In general, we observed stronger effects of oxalyl surfen than surfen (Fig. 2).

### Surfen and oxalyl surfen rescue motoneuron defects and behavioral deficits

**Fig. 3:**
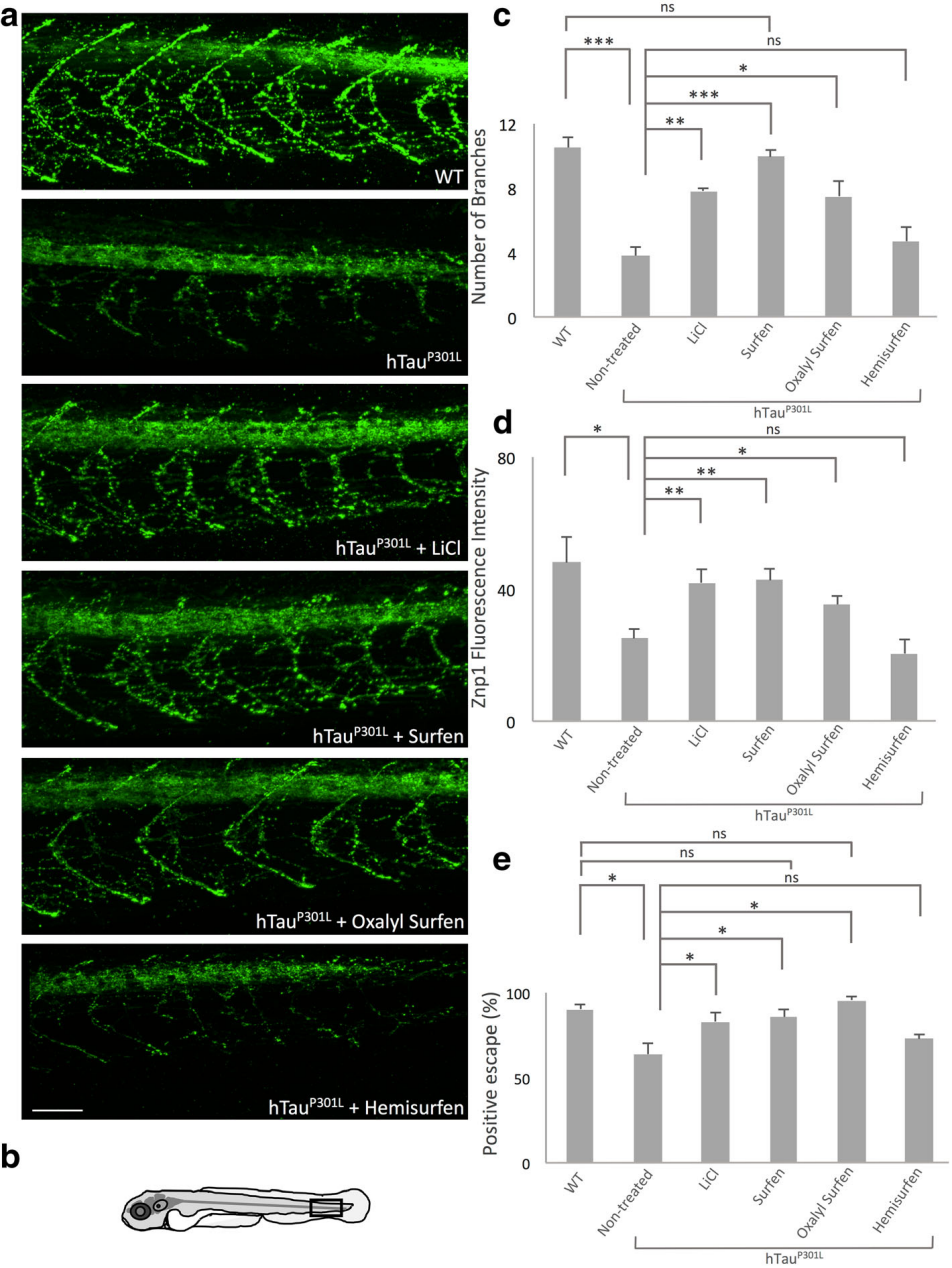
Surfen and oxalyl surfen rescue motoneuron defects and functional deficits in Tg[HuC::hTau^P301L^; DsRed] embryos. **a** Immunohistochemical visualization of the synaptotagmin II protein (anti-znpl antibody) in 72 hpf wild-type (WT) and Tg[HuC::hTau^P301L^; DsRed] (hTau^P301L^) embryos treated for 2 days with 1% DMSO (hTau^P301L^ + 1% DMSO), 80 mM LiCl (hTau^P301L^ + LiCl), 3 μM surfen (hTau^P301L^ + surfen), 2 μM oxalyl surfen (hTau^P301L^ + oxalyl surfen) or 3 μM hemisurfen (hTau^P301L^ + hemisurfen), showed that surfen and oxalyl surfen markedly rescued the motorneuron axon defects seen in Tg[HuC::hTau^P301L^; DsRed] embryos. The imaged caudal area corresponds to the box represented in (**b**). Scale bar: 50 μm. **c** Quantification of the mean axonal branch number in the framed caudal area (**b**) of Tg[HuC::hTau^P301L^; DsRed] embryos treated as in (**a**). The number of axonal branches was significantly increased in Tg[HuC::hTau^P301L^; DsRed] embryos treated with surfen and oxalyl surfen when compared with their age-matched 1% DMSO siblings (*n* = 10, ***P < 0.001; **P < 0.01; *P < 0.05; ns: non-significant, Student’s *t* test). **d** Quantification of fluorescence staining intensity of znp1 labeling in the framed caudal area (**b**) of Tg[HuC::hTau^P301L^; DsRed] embryos treated as in (**a**). Results showed that the fluorescence intensity of the znp1 staining was significantly increased in Tg[HuC::hTau^P301L^; DsRed] embryos following surfen and oxalyl surfen treatments when compared with their age-matched 1% DMSO siblings (*n* = 10, **P < 0.01; *P < 0.05, ns: non-significant, Student’s *t* test). **e** The touch evoked escape response was used to probe the motor behavior of Tg[HuC::hTau^P301L^; DsRed] embryos treated for 1 day with 80 mM LiCl, 3 μM surfen, 2 μM oxalyl surfen or 3 μM hemisurfen. The motor defect observed in Tg[HuC::hTauP301L; DsRed] embryos was significantly rescued following treatment with surfen and oxalyl surfen, but not hemisurfen (*n* = 250, *P < 0.05, ns: non-significant, Student’s *t* test)

Tg[HuC::hTau^P301L^; DsRed] larvae displayed markedly reduced motoneuron axon-branching and elongation, and, as a likely consequence, they showed an impaired escape response to touch stimuli [3]. Because surfen and oxalyl surfen markedly decrease tau hyperphosphorylation, we next investigated whether treatment with these two compounds could rescue, at least partially, neuronal deficits and promote functional recovery. First, we analysed motoneuron axon morphology by immunohistochemistry using znp1 [17], an antibody that specifically recognizes synaptotagmin II, a synaptic protein highly expressed in primary motoneuron axons. Results showed that treatment with surfen, oxalyl surfen, and LiCl, but not hemisurfen, significantly increased primary motoneuron branching in Tg[HuC::hTau^P301L^, DsRed] embryos (1% DMSO vs. surfen-treated embryos: *P* < 0.001; 1% DMSO vs. oxalyl surfen-treated embryos: *P* < 0.05; 1% DMSO vs. LiCl-treated embryos: *P* < 0.01; 1% DMSO vs. hemisurfen-treated embryos: *P* = 0.43) (Fig. 3a and b).

Next, we quantified znp1 staining in wild-type and in treated and 1% DMSO Tg[HuC::hTau^P301L^; DsRed] embryos. As previously shown [3], a significant decrease in znp1 staining was observed in 1% DMSO Tg[HuC::hTau^P301L^, DsRed] individuals when compared to age-matched wild-type embryos (*P* < 0.05) (Fig. 3a and c). Interestingly, znp1 staining in Tg[HuC::hTau^P301L^; DsRed] embryos was significantly rescued following a 2 day treatment with surfen (*P* < 0.01), oxalyl surfen (*P* < 0.05), and LiCl (*P* < 0.01), but not hemisurfen (*P =* 0.21) (Fig. 3a and c).

We next assessed whether treatment with surfen or oxalyl surfen could rescue the motility defects of Tg[HuC::hTau^P301L^; DsRed] zebrafish larvae in response to touch stimuli. As previously shown, Tg[HuC::hTau^P301L^; DsRed] zebrafish larvae showed significantly impaired motility characterized by slower movements and reduced touch-induced escape when compared to wild-type age-matched larvae (wild-type vs. 1% DMSO Tg[HuC::hTau^P301L^; DsRed] embryos: *P* < 0.05) (Fig. 3d). Interestingly, treatment with surfen or oxalyl surfen, fully rescued the motility deficit (surfen and oxalyl surfen vs. 1% DMSO: *P* < 0.05; Fig. 3d), while treatment with hemisurfen had no effect on motility defects (*P* = 0.27; Fig. 3d). These results provided functional evidence that surfen and oxalyl surfen not only significantly decrease tau hyperphosphorylation, but also alleviate the behavioural consequences of the neuronal deficits induced by the expression of the human mutant tau^P301L^ protein.

## Discussion

Here we showed that treatment with surfen, or its analog, oxalyl surfen, significantly decreased tau hyperphosphorylation and rescued motoneuron defects and behavioral abnormalities induced by expression of mutant hTau^P301L^ in Tg[HuC::hTau^P301L^; DsRed] zebrafish. In contrast, hemisurfen did not affect tau phosphorylation, motoneuron and behavioural deficits. Surfen has been shown to inhibit heparan sulfate-protein interactions [13], whereas hemisurfen does not [14]. Thus surfen and oxalyl surfen may mediate their beneficial effects by blocking heparan sulfate-tau interactions. Taken together, these results strengthen the hypothesis of a substrate modulator effect of highly sulfated HS chains on tau, possibly through a chaperone-like activity uncovering the tau residues that are phosphorylated in pathological situations. In this context, surfen and oxalyl surfen may inhibit the chaperone activity of sulfated polysaccharide chains, thus preventing tau hyperphosphorylation. It is also possible that the decline in tau phosphorylation mediated by surfen and oxalyl surfen is due to alterations of heparan sulfate biosynthesis or metabolism. Surfen may affect heparan sulfate structure and stability, as it has been previously shown in vitro that surfen prevents interaction of heparin with heparin biosynthetic and degrading enzymes (Schuksz et al.). However, it should also be noted that surfen also displays Ca channel blocker [18], zinc ion binding [11] and immunomodulatory activities [10, 19]. These properties can potentially affect pathophysiological mechanisms in tauopathies. Calcium deregulation is reported to contribute to neurodegeneration in iPSC-derived neurons from FTD patients [20]. Surfen may affect calcium levels by blocking calcium channels or modulating calcium channels through interaction with heparan sulfate [21]. Zinc ion (Zn^2+^) has been implicated in tau fibrillization and toxicity [22, 23]. A c5a receptor antagonist is reported to decrease tau hyperphosphorylation in 3xTg mouse model of Alzheimer’s disease [24], while surfen also acts as an inhibitor of c5a receptor binding [10].

Importantly, apart from rare reports of hypersensitivity reactions [25, 26], surfen is well tolerated in clinical settings. Although one study linked high doses and prolonged administration of surfen to lymphosarcoma and lesions reminiscent of nutritional deficiency, surfen is well tolerated in mice [13]. In good agreement, we found surfen and oxalyl surfen to be well tolerated in zebrafish embryos at the effective concentrations of 3 μM and 2 μM, respectively. Oxalyl surfen shows a stronger effect on tau phosphorylation and behavioural rescue than surfen, suggesting the molecule to be more efficient against tau pathology. However, the derivative is toxic to zebrafish embryos at a lower concentration, pointing out a potentially less favourable safety profile than surfen. Further investigations are needed to determine whether the two molecules could also counteract neurodegenerative processes linked to tau alterations in humans.

## Materials and methods

### Animals

Zebrafish were maintained at 28 °C in our zebrafish facility under standard conditions as described by Westerfield (1995) [27]. Developmental stages were determined as hours post-fertilization (hpf) as described by Kimmel et al. [28]. *AB* strain was used as wild-type fish. The zebrafish transgenic line stably expressing the human mutant Tau^P301L^ protein that is associated with frontotemporal dementia with Parkinsonism linked to chromosome 17 (FTDP-17) (the Tg[HuC::hTau^P301L^; DsRed]), has been previously described [3], and was kindly provided by Christian Haass, Bettina Schmid, and Dominik Paquet (Deutsches Zentrum für Neurodegenerative Erkrankungen or DZNE, Munich, Germany).

### Compounds

Surfen (1,3-bis(4-amino-2-methylquinolin-6-yl)urea) was obtained from the Open Chemical Repository in the Developmental Therapeutic Program at the National Cancer Institute (NSC12155) or synthesized according to published methods. The synthesis and characterization of oxalyl surfen (N1,N2-bis(4-amino-2-methylquinolin-6-yl)oxalamide) and hemisurfen (1-(4-amino-2-methylquinolin-6-yl)urea) have been previously described [14, 29].

### Treatments

As surfen and oxalyl surfen bind avidly to plastic, we either pre-coated all plasticware with serum containing medium or used glass vessels. Stock solutions (surfen and hemisurfen, 30 mM; oxalyl surfen, 21.7 mM) were prepared in DMSO. Working solutions were prepared as needed by diluting stock solutions in E3 medium and adjusting the DMSO concentration to 1% (vol/vol). Final concentrations for treatments were determined as 3 μM for surfen, 2 μM for oxalyl surfen, 3 μM for hemisurfen and 80 mM for lithium chloride (LiCl) based on maximal non-toxic concentrations for zebrafish embryos (Additional file 1). Embryos (24 hpf) were manually dechorionated and incubated for 2 days in 1–2 ml of either control medium (E3 medium containing 1% DMSO) or E3 medium containing 1% DMSO and the surfen derivatives in BSA-coated 6-well microtiter plates. All solutions were changed daily.

### ELISA

Embryos treated as previously described were anaesthetized with MS-222 in E3 medium on ice. After removal of the yolk, embryos were snap frozen on dry ice and homogenized by sonication in lysis buffer (50 mM Tris HCl, 150 mM NaCl, 10% Triton X100, 1 mM EDTA, 1X Protease Inhibitor Cocktail [Roche], 1 mM Sodium orthovanadate (NaVO_4_) [Sigma-Aldrich], and 1 mM Sodium fluoride (NaF), pH 8). Insoluble material was removed by a 30 min centrifugation (10,000 *g*) at 4 °C and protein concentration was determined with Bradford protein assay (Bio-Rad). Accumulation of phosphorylated tau was quantified using the INNOTEST® Phospho-Tau (181P) ELISA (Innogenetics, Gent Belgium), using mAb HT7 for coating, phospho-dependent mAb AT270 (specific for phospho-threonine-181 tau epitope) as detector antibody, and a synthetic phosphopeptide for standardization.

### Western blot

Zebrafish larvae were collected, anaesthetized in MS-222, and lysed on ice with lysis buffer (50 mM Tris-HCl, 150 mM NaCl, 1% Triton X-100, 10 mM NaF, 1 mM Na_3_VO_4_, pH 8.0) supplemented with protease and phosphatase inhibitors (Pierce). Lysates were homogenized by sonication and centrifuged at 12000 *g* for 15 min. The protein content in the supernatants was quantified using a Bradford protein assay (Bio-Rad).

Samples containing 10 μg proteins were subjected to SDS-PAGE in 10% acrylamide gel. Primary antibodies against phosphorylated tau, AT270 and PHF1 (Pierce, Thermo Scientific), anti-human total tau antibody K9JA (Rabbit Polyclonal Antibody, Dako Cytomation), and anti-GADPH (Abcam) were used. Blots were subsequently incubated for 1 h at room temperature with the corresponding secondary antibodies diluted in phosphate-buffered saline containing 5% milk and revealed using ECL RevelBlOt® Plus (Ozyme) following manufacturer’s instructions.

### Immunohistochemistry

For immunohistochemical analysis, embryo pigmentation was inhibited by treatment with 0.2 mM 1-phenyl-2-thiourea (PTU) in E3 medium starting at 20 hpf. After anaesthesia with MS-222, whole embryos were fixed in 4% paraformaldehyde in PBS, and preserved in methanol (MeOH) 100%. Fixed and acetone-cracked embryos were then blocked and permeabilized for 1 h at room temperature in a PBS solution containing 10% NGS, 1% DMSO and 0.1% Tween 20. Embryos were then incubated overnight at room temperature with the phosphorylation-independent primary anti-human total tau antibody K9JA (Rabbit Polyclonal Antibody, Dako Cytomation) diluted at 1:300, the phosphorylation-dependent site specific anti-PHF-Tau antibody AT8 diluted at 1:50 (gift from D. Paquet), and anti-Znp-1 (Mouse Monoclonal Antibody; Hybridoma Bank, Iowa, USA) (1:300) to investigate the morphology of primary motoneurons. After several washes, embryos were blocked as previously described [30], and incubated overnight at 4 °C with a solution containing CY3-coupled goat anti-rabbit (1:500) and Alexa Fluor 488-coupled goat anti-mouse antibody (1:500). Embryos were mounted in 1% agarose (low melting, Bio-Rad) in PBS buffer.

### Image analysis

Bright field images of embryos were captured using a stereomicroscope (SteREO Lumar. V12, Zeiss) equipped with a digital camera (DXM 1200F, Nikon) controlled by the ACT-1 software (Version 2.63 Nikon). Fluorescently labelled embryos were imaged using a microscope equipped with an ApoTome system (Zeiss) fitted with an AxioCam MRm camera (Zeiss) controlled by the Axiovision or ZEN software. All images were processed with Adobe Photoshop 7.0 (Adobe System, San Jose, CA). When necessary, brightness, contrast, and colour balance, were uniformly optimized. Fluorescence intensities and densimetric quantification of protein immunoblots were performed using ImageJ/Fiji (Rasband, W.S., ImageJ, U. S. National Institutes of Health, Bethesda, Maryland, USA, http://imagej.nih.gov/ij/, 1997–2012) on grayscale images. For each value, quantifications were performed using images from three independent experiments.

### Behavioural analysis

Larvae behaviour was analysed at 48 hpf after 1 day treatments with the different compounds. The larval escape response reflex was assessed by gently touching the tip of the tail with a fine plastic rod. Embryos were classified as responders or non-responders, with non-responders failing to respond by swimming at least three times their own body length.

### Statistics

Values for mean, standard deviation (SD) and standard error of mean (SEM) were calculated using Microsoft Excel, version 12.0.6683.5002. Statistical analysis was performed using Microsoft Excel and Student’s *t* test. Error bars represent SEM, *P < 0.05, ** *P <* 0.01, ***P < 0.001.

## Additional file

Additional file 1: **Figure S1.** Percentage of embryonic survival observed for 72 hpf wild-type (WT) and Tg[HuC::hTauP301L; DsRed] (non-treated) embryos incubated for 2 days in E3 medium containing 1% DMSO or E3 medium containing 1% DMSO with LiCl (10–150 mM) **(a)**, surfen (0.1–10 μM) **(b)**, oxalyl surfen (0.1–10 μM) **(c)** or hemisurfen (0.1–10 μM) **(d)**. Note that at the selected concentrations (80 mM LiCl, 3 μM for surfen and hemisurfen and 2 μM for oxalyl surfen) are the maximal non-toxic concentrations (*n* = 250, *P < 0.05, ***P < 0.001, ns: non-significant, Student’s *t* test). (TIFF 55462 kb)

## Abbreviations

AD: Alzheimer’s disease
DMSO: Dimethyl sulfoxide
dpf: Days post fertilization
FTD: Frontotemporal dementia
hpf: Hours post fertilization
LiCl: Lithium chloride

## Authors’ information

SMAN, CY, SB and NSY are from the Département Hospitalo-Universitaire: Promoting Research Oriented Towards Early CNS Therapies, Inserm, Hôpital Robert Debré, Paris, France. The present address for SMAN is Laboratoire Neurosciences Paris Seine, INSERM UMRS 1130, UMR 8246, Université Pierre et Marie Curie, 75252 Paris, France. MB is from the Department of Biology, Cadi Ayyad University, Marrakesh, Morocco. RJW and YT are affiliated with the Department of Chemistry and Biochemistry, University of California, San Diego, La Jolla, CA, USA. JDE is from the Department of Cellular and Molecular Medicine, University of California, San Diego, La Jolla, CA, USA.
