# Correction to: Surfen and oxalyl surfen decrease tau hyperphosphorylation and mitigate neuron deficits in vivo in a zebrafish model of tauopathy

**DOI:** 10.1186/s40035-020-00220-3

**Published:** 2020-12-20

**Authors:** Seyedeh Maryam Alavi Naini, Constantin Yanicostas, Rahma Hassan-Abdi, Sébastien Blondeel, Mohamed Bennis, Ryan J. Weiss, Yitzhak Tor, Jeffrey D. Esko, Nadia Soussi-Yanicostas

**Affiliations:** 1 PROTECT, Inserm, Université Paris Diderot, Sorbonne Paris Cité, Paris, France; 2 Institut de Biologie Paris Seine-Laboratoire Neuroscience Paris Seine, Inserm UMRS 1130, CNRS UMR 8246, UPMC UM 118, Université Pierre et Marie Curie, Paris, France; 3 Cadi Ayyad University, Marrakesh, Morocco; 4 Department of Chemistry and Biochemistry, University of California, San Diego, La Jolla, CA USA; 5 Department of Cellular and Molecular Medicine, University of California, San Diego, La Jolla, CA USA

**Correction to: Transl Neurodegener 7, 6 (2018)**

**https://doi.org/10.1186/s40035-018-0111-2**

Following the publication of the original article [1], it was noted that due to a typesetting error several lines are mistakenly added in the Fig. 1 and they should be deleted.

**Fig. 1:**
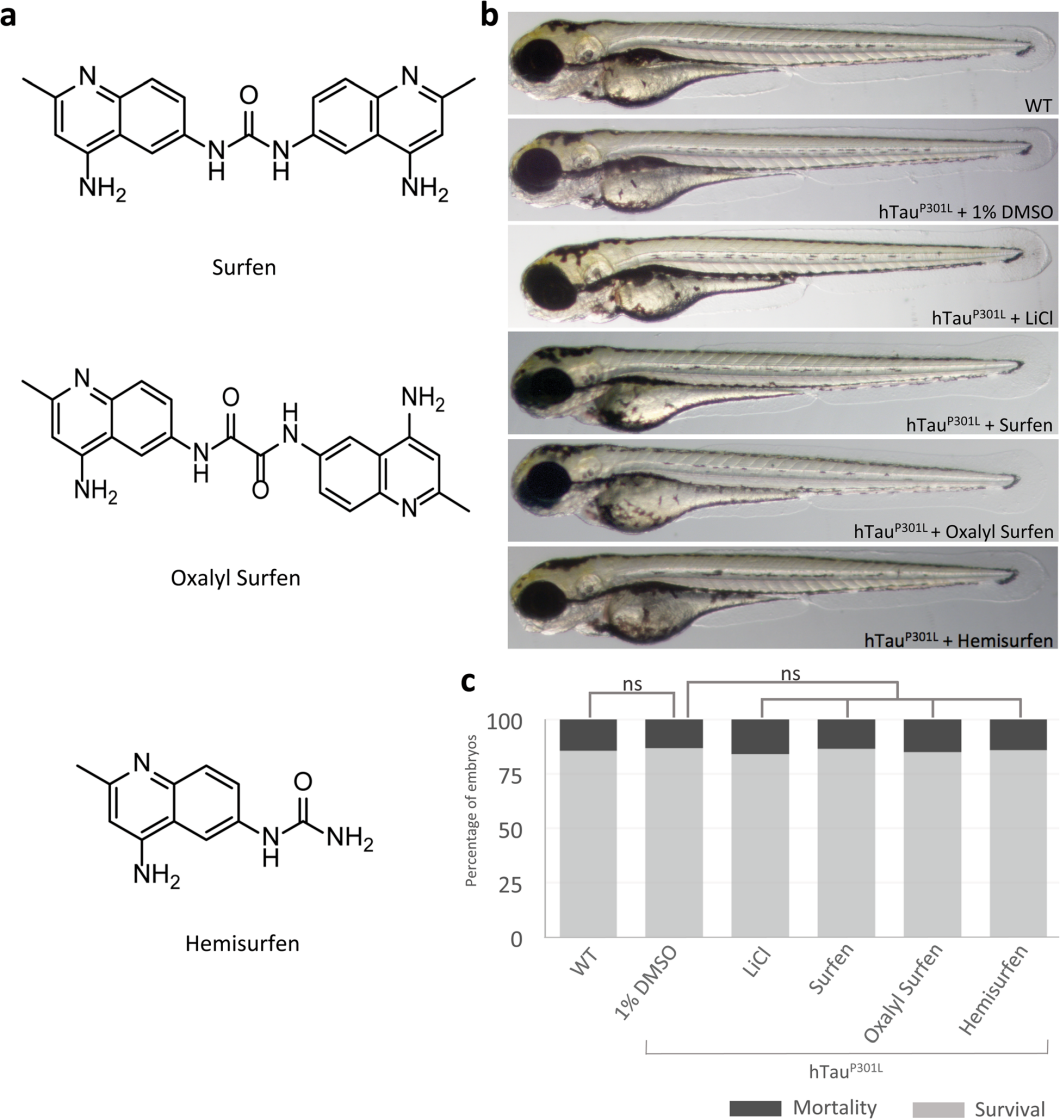
Surfen and oxalyl surfen are well tolerated by Tg [HuC::hTau^P301L^; DsRed] embryos. **a** Chemical structure of Surfen (1,3-bis(4-amino-2-methylquinolin-6-yl) urea), oxalyl surfen (N1,N2- bis(4-amino-2-methylquinolin-6-yl)oxalamide) and hemisurfen (1-(4-amino-2-methylquinolin-6- yl)urea). **b** Phenotypic analysis of 72 hpf wild-type (WT) and Tg [HuC::hTau^P301L^; DsRed] (hTau^P301L^) embryos incubated for 2 days in E3 medium containing 1% DMSO (hTau^P301L^ + 1% DMSO), 80 mM LiCl (hTau^P301L^ + LiCl), 3 μM surfen (hTau^P301L^ + surfen), 2 μM oxalyl surfen (hTau^P301L^ + oxalyl surfen) or 3 μM hemisurfen (hTau^P301L^ + hemisurfen), showed that embryonic development is not impaired by the treatments. Magnification × 40. **c** Survival rate of 72 hpf wild-type (WT) and Tg [HuC::hTau^P301L^; DsRed] (hTau^P301L^) embryos incubated for 2 days in E3 medium containing 1% DMSO (hTau^P301L^ + 1% DMSO), 80 mM LiCl (hTau^P301L^ + LiCl), 3 μM surfen (hTau^P301L^ + surfen), 2 μM oxalyl surfen (hTau^P301L^ + oxalyl surfen), or 3 μM hemisurfen (hTau^P301L^ + hemisurfen), demonstrated that embryonic mortality was not significantly increased by treatments (*n* = 250, *P >* 0.05, Student’s t test)

The correct figure has been included in this correction, and the original article has been corrected.
