# Supplementary figures and images for: Surfen and oxalyl surfen decrease tau hyperphosphorylation and mitigate neuron deficits in vivo in a zebrafish model of tauopathy
Source: Transl Neurodegener. 2018 Mar 16;7:6. doi: 10.1186/s40035-018-0111-2 (PMC5855975; doi:10.1186/s40035-018-0111-2)

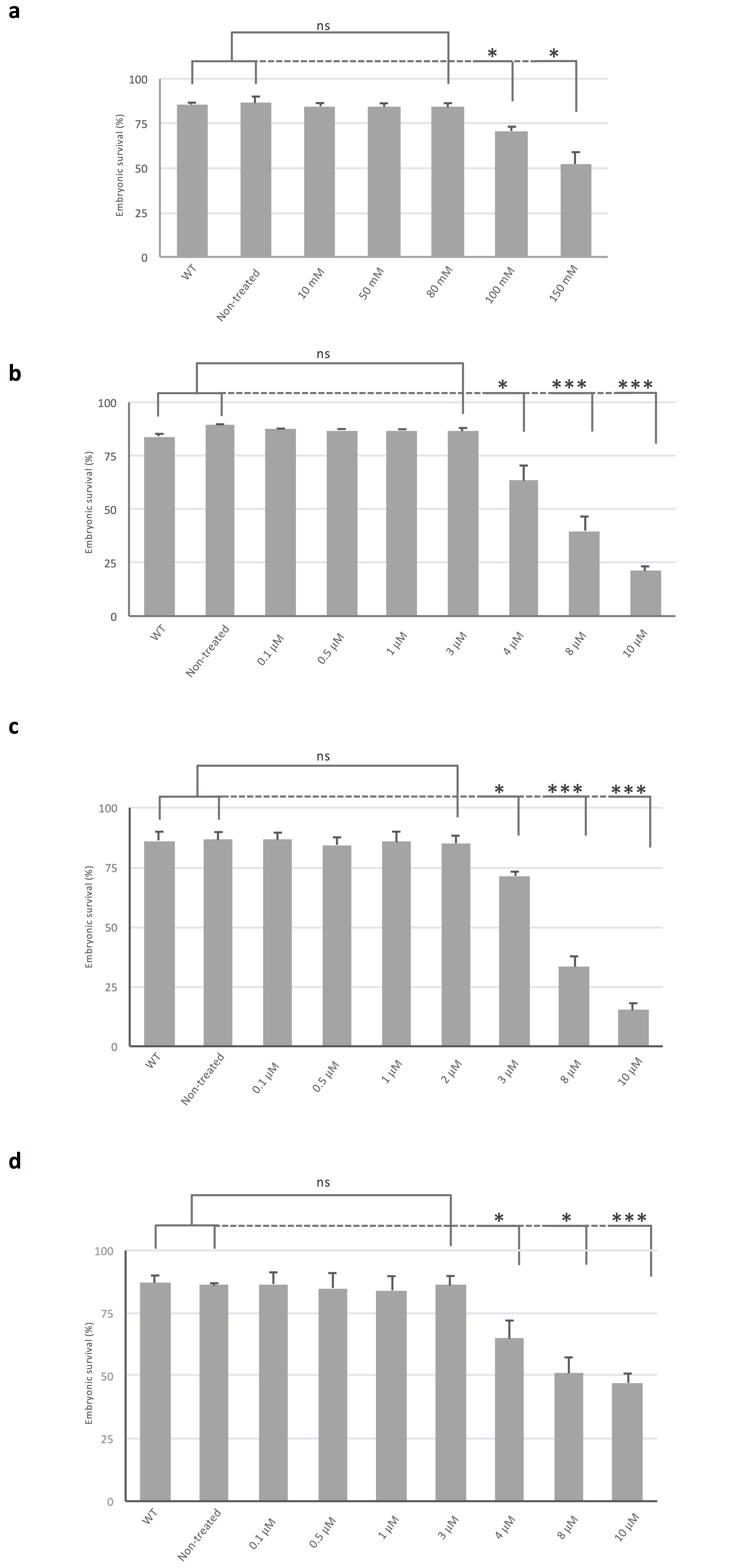

Supplement: Supplementary file 1 — Figure S1. Percentage of embryonic survival observed for 72 hpf wild-type (WT) and Tg[HuC::hTauP301L; DsRed] (non-treated) embryos incubated for 2 days in E3 medium containing 1% DMSO or E3 medium containing 1% DMSO with LiCl (10–150 mM) (a), surfen (0.1–10 μM) (b), oxalyl surfen (0.1–10 μM) (c) or hemisurfen (0.1–10 μM) (d). Note that at the selected concentrations (80 mM LiCl, 3 μM for surfen and hemisurfen and 2 μM for oxalyl surfen) are the maximal non-toxic concentrations (n = 250, *P < 0.05, ***P < 0.001, ns: non-significant, Student’s t test). (TIFF 55462 kb) [file 40035_2018_111_MOESM1_ESM.tiff]
